# Co-occurring conditions in children with Down syndrome and autism: a retrospective study

**DOI:** 10.1186/s11689-023-09478-w

**Published:** 2023-03-02

**Authors:** Noemi A. Spinazzi, Jonathan D. Santoro, Katherine Pawlowski, Gabriel Anzueto, Yamini J. Howe, Lina R. Patel, Nicole T. Baumer

**Affiliations:** 1 UCSF Benioff Children’s Hospital Oakland, Oakland, CA 94609 USA; 2 Division of Neuroimmunology, Department of Pediatrics, Children’s Hospital Los Angeles, Los Angeles, CA 90027 USA; 3 Department of Neurology, Keck School of Medicine, University of Southern California, Los Angeles, CA 90033 USA; 4 Division of Developmental Medicine, Boston Children’s Hospital, Boston, MA 02115 USA; 5 Division of Developmental and Behavioral Pediatrics, Ann & Robert H. Lurie Children’s Hospital of Chicago, Chicago, IL 60611 USA; 6 Feinberg School of Medicine, Northwestern University, Chicago, IL 60611 USA; 7 Massachusetts General Hospital Lurie Center for Autism, Lexington, MA 02421 USA; 8 Harvard Medical School, Boston, MA 02115 USA; 9 Department of Psychiatry, Children’s Hospital Colorado, Aurora, CO 80045 USA; 10 Department of Neurology, Boston Children’s Hospital, Boston, MA 02115 USA

**Keywords:** Down syndrome, Autism spectrum disorder, Medical disease, Comorbid, Prevalence, Co-occurring medical conditions

## Abstract

**Background:**

Down syndrome (DS) is one of the most common genetic causes of intellectual disability, and it is associated with an increased incidence of numerous co-occurring conditions. Autism spectrum disorder (ASD) is common in persons with DS, with rates reported as high as 39%. However, little is known regarding co-occurring conditions in children with both DS and ASD.

**Methods:**

A single-center retrospective review of prospective longitudinally collected clinical data was performed. Any patient with a confirmed diagnosis of DS evaluated at a large, specialized Down Syndrome Program in a tertiary pediatric medical center between March 2018 and March 2022 was included. A standardized survey which included demographic and clinical questions was administered during each clinical evaluation.

**Results:**

In total, 562 individuals with DS were included. The median age was 10 years (IQR: 6.18–13.92). Of this group, 72 (13%) had a co-occurring diagnosis of ASD (DS+ASD). Individuals with DS+ASD were more likely to be male (OR 2.23, CI 1.29–3.84) and had higher odds of a current or prior diagnosis of constipation (OR 2.19, CI 1.31–3.65), gastroesophageal reflux (OR 1.91, CI 1.14–3.21), behavioral feeding difficulties (OR 2.71, CI 1.02–7.19), infantile spasms (OR 6.03, CI 1.79–20.34) and scoliosis (OR 2.73, CI 1.16–6.40). There were lower odds of congenital heart disease in the DS+ASD group (OR 0.56, CI 0.34–0.93). There was no observed difference in prematurity or Neonatal Intensive Care Unit complications between groups. Individuals with DS+ASD had similar odds of having a history of congenital heart defect requiring surgery to those with DS only. Furthermore, there was no difference in rates of autoimmune thyroiditis or celiac disease. There was also no difference in rates of diagnosed co-occurring neurodevelopmental or mental health conditions in this cohort, including anxiety disorders and attention-deficit/hyperactivity disorder.

**Conclusions:**

This study identifies a variety of medical conditions which are more frequent in children with DS+ASD than DS alone, providing important information for the clinical management of these patients. Future research should investigate the role of some of these medical conditions in the development of ASD phenotypes, and whether there may be distinct genetic and metabolic contributions towards these conditions.

**Supplementary Information:**

The online version contains supplementary material available at 10.1186/s11689-023-09478-w.

## Background

Autism spectrum disorder (ASD) is increasingly recognized in individuals with Down syndrome (DS). Meta-analysis data suggests that 16–18% of individuals with DS also have ASD [1], with estimates from individual studies using differing ascertainment criteria ranging from 5 to 39% [2–6]. There is a growing need to better understand the risk factors and health needs of children with a dual diagnosis of DS and ASD (DS+ASD).

DS presents with variable cognitive and physical phenotypes, and it is associated with increased prevalence of a wide range of medical conditions [7–10], including premature birth, congenital heart disease (CHD), congenital and acquired thyroid disease, autoimmune disease, congenital and functional gastrointestinal (GI) pathology, sleep disturbances including sleep apnea, and seizure disorders including infantile spasms. Individuals with idiopathic ASD are also at higher risk for several medical conditions [11–13], including constipation and feeding difficulties, epilepsy, and sleep disorders. Additionally, conditions that commonly occur in DS, such as CHD and prematurity, have been shown to proffer an increased risk of ASD in the general population [14, 15]. While determination of key features and phenotypic presentation of DS+ASD has advanced over the past decade, evaluation of risk factors and occurrence of medical conditions in this group of individuals lags behind. To date, few studies have attempted to identify medical, mental health (MH), and neurodevelopmental (ND) co-occurrences in individuals with DS+ASD; some lacked a control group [16], while others had small sample sizes [17, 18].

This study investigated the prevalence of medical, MH, and ND conditions in individuals with DS+ASD compared to those with DS alone.

## Methods

### Overview

The Boston Children’s Hospital Down Syndrome Program (BCH DSP) established a standardized clinical data collection process and database in March of 2018. As part of routine clinical care, prior to each visit, caregivers complete standardized intake forms including past and present medical history. After each clinical visit, clinicians complete a data form indicating diagnostic and treatment information of co-occurring ND and MH conditions. Starting in July 2021, the DSP clinicians began completing a clinician-verified history form that covered all body systems. Patients whose visits occurred between March 2018 and July 2021 have caregiver-provided past and present medical history information available; those seen from July 2021 to March 2022 had both caregiver-provided and clinician-verified medical history data. All patients had clinician-verified ND/MH information available. Clinicians and caregivers designated each condition as “not present,” “current/ongoing,” or “past/resolved.” A detailed description of database infrastructure, database methodology, and data forms was previously reported [19]. This study analyzing the clinically-collected data was approved by the Institutional Review Board.

### Participants

Patients with confirmed DS who were seen in the DSP between March 2018 and March 2022 (*N* =858), and who had any portion of the clinical intake forms completed were included in the database. Out of the 858 patients, 692 unique patients had completed caregiver-provided medical history information and had a clinician-confirmed categorization of ASD or no ASD. ASD was diagnosed by expert clinicians using the Diagnostic Statistical Manual (DSM)- 5 diagnostic criteria, with particular attention to criterion E, which requires that symptoms not be due to intellectual disability or global developmental delay. Clinician diagnostic certainty has been shown to be the best predictor of ASD [20], and according to a recently published systematic review [21], the DSM is the diagnostic criteria most used as a reference in diagnosing ASD in patients with DS [20]. In this cohort, only 29 patients had ever been evaluated with the Autism Diagnostic Observation Schedule (ADOS), with detailed results only available for 4 individuals. Analysis was restricted to patients 3 years and older (*N*= 562) for several reasons: no children younger than 3 in the sample were given a diagnosis of ASD; it is not yet clear that ASD can be reliably diagnosed clinically in toddlers with DS and co-existing developmental delays [4] (unlike children without DS, in whom ASD can generally be diagnosed by 18–24 months of age) [22]; and ASD is often diagnosed later in children with DS [21].

### Procedures

Information generated from clinical visits was stored and managed using Research Electronic Data Capture (REDCap) [23, 24]. The REDCap database is protected for study subjects’ privacy and is fully compliant with HIPAA regulations. Information was abstracted from the Database and the Electronic Medical Record (EMR) system for three key categories: (1) socio-demographic information (EMR), (2) birth history and medical conditions (Database), and (3) neurodevelopmental and mental health conditions (Database).

### Sociodemographic information

Sex, race, primary language spoken at home and zip code were all caregiver-reported demographic fields collected from the EMR. Income quartiles were estimated using the median household income per patient zip code [25].

### Birth history and medical conditions

Birth history included frequency counts for primary diagnosis, timing of diagnosis, gestational age, length of stay greater than 4 days in the hospital/Neonatal Intensive Care Unit (NICU), and perinatal complications. Gestational age was divided into 2 sub-categories: preterm (less than 37 weeks) and full term (37 weeks or more). Any medical diagnosis endorsed by caregivers as either “past/resolved” or “current/ongoing” was combined into an “ever diagnosed” category. Some medical conditions were also grouped into composite variables for additional analysis, specifically: CHD with and without a history of surgery; GI problems; and epilepsy (Additional file 1). Age was defined as the age at the most recent clinical visit within the data collection timeframe (between March 2018 and March 2022).

### Neurodevelopmental and mental health conditions

Any confirmed diagnosis of a MH condition (anxiety, depression, obsessivecompulsive disorder or regression/catatonia) or ND disorder (ASD or attention-deficit/ hyperactivity disorder (ADHD)) was also combined into an “ever diagnosed” category, if described by a clinician as either “past/resolved” or “current/ongoing.”

### Statistical analysis

Demographics and medical information were reported as counts and percentages related to the total participant population of either DS alone or DS+ASD (*N*). As information about each individual condition was not available for 100% of the sample, the sample sizes were different for each medical and neurodevelopmental condition. A student’s *T*-test was used to determine whether there was a significant difference in the age distributions between the two groups, and Cohen’s D_s_ was calculated to determine effect size. Odds ratios (OR) were performed to determine the relative risk of the contribution of medical conditions towards a diagnosis of DS+ASD. Corresponding p-values with 95% confidence intervals were calculated for each odds ratio. A two-sided p-value of 0.05 was used to determine statistical significance.

## Results

Demographic data is presented in Table 1. Most patients in the sample were White and English-speaking, with no difference between the DS and DS+ASD groups. The median age of individuals in the sample was 10 years old (interquartile range (IQR): 6.18–13.92), and the mean age was 10.48. Patients in the DS+ASD cohort were older, with a mean age of 11.74 versus 10.19 years in the DS-only group. This difference was statistically significant (*p*=0.01), though the actual difference is a little more than a year, with a small to medium effect size (Cohen’s D_s_ = 0.31).

**Table 1:** Population demographics

	All (***N***=562)	DS+ASD (***N***=72)	DS-only (***N***=490)
**Sex (*n*, %)**			
**Male**	316 (56.2%)	52 (72.2%)	264 (53.9%)
**Diagnosis (*n*, %)**			
**Trisomy 21**	541 (96.3%)	66 (91.7%)	475 (96.9%)
**Mosaic**	8 (1.4%)	4 (5.6%)	4 (0.8%)
**Translocation**	10 (1.8%)	1 (1.4%)	9 (1.8%)
**Unknown**	3 (0.5%)	1 (1.4%)	2 (0.5%)
**Age (years)**			
**Median**	10	11	10
**IQR**	7.74	8.14	7.47
**Mean**	10.39	11.74	10.19
**Standard deviation**	5.04	5.06	5.02
**Race/ethnicity (*n*, %)**			
**White**	382 (68.0%)	46 (63.9%)	336 (68.6%)
**Black**	42 (7.5%)	5 (6.9%)	37 (7.6%)
**Asian**	20 (3.6%)	2 (2.8%)	18 (3.8%)
**Native Alaskan/American Indian**	0 (0%)	0 (0%)	0 (0%)
**Pacific Islander/Native Hawaiian**	1 (0.2%)	1 (1.4%)	0 (0%)
**Multiple races reported**	1 (0.2%)	0 (0%)	1 (0.2%)
**Other**	68 (12.1%)	13 (18.1%)	55 (11.2%)
**No answer**	48 (8.5%)	5 (6.9%)	43 (8.8%)
**Primary language (*n*, %)**			
**English**	514 (91.5%)	69 (95.8%)	445 (90.8%)
**Spanish**	24 (4.3%)	2 (2.8%)	22 (4.5%)
**Portuguese**	8 (1.4%)	1 (1.4%)	7 (1.4%)
**Other**	14 (2.5%)	0 (0%)	13 (2.7%)
**No answer**	3 (0.5%)	0 (0%)	3 (0.6%)
**Annual household income (*n*, %)**			
**< $34,282**	28 (5.0%)	4 (5.6%)	24 (4.5%)
**$34,282–$54,850**	217 (38.6%)	29 (40.3%)	188 (38.4%)
**$54,850–$82,276**	245 (43.6%)	33 (45.8%)	212 (43.3%)
**>$82,276**	70 (12.5%)	6 (8.3%)	64 (13.1%)
**No answer**	2 (3.6%)	0 (0%)	2 (0.4%)

Medical comorbidities are presented in Table 2. Individuals with a clinician-confirmed ASD diagnosis were twice as likely to be male. Compared to individuals with DS alone, the prevalence of GI problems (composite variable, OR: 2.18, *p*=0.008, 95% CI: 1.23–3.87), and specifically constipation (OR: 2.19, *p*=0.003, 95% CI: 1.31–3.65) and gastroesophageal reflux (OR: 1.91, *p*=0.01, 95% CI: 1.14–3.21) were more frequent in individuals with DS+ASD. Furthermore, individuals with DS+ASD were more likely to have behavioral feeding difficulties (OR: 2.71, *p*=0.04, 95% CI: 1.02–7.19). There was a higher prevalence of scoliosis in the DS+ASD group (OR: 2.73, *p*=0.02 95% CI: 1.16–6.40). Individuals with DS+ASD had significantly higher odds of having a history of epilepsy (OR: 2.70, *p*=0.046, 95% CI: 1.02–7.17) and infantile spasms (OR: 6.03, *p*=0.004, 95% CI: 1.79–20.34). Conversely, individuals with DS alone were more likely to have a history of CHD (composite variable, OR: 0.56, *p*=0.02, 95% CI: 0.34–093) and specifically atrial septal defects (OR: 0.36, *p*=0.009, 95% CI: 0.17–0.78).

**Table 2:** Rates of medical, neurodevelopmental, and psychiatric co-morbidities of study participants

Category	All (***n***/***N***, %)	DS+ASD (***n***/***N***, %)	DS-only (***n***/***N***, %)	Odds ratio	***p*** value (95% CI)
**Male sex**	316/562 (56.2%)	52/72 (72.2%)	264/490 (53.9%)	2.23 ^a^	0.004 (1.29–3.84)
**English as primary language**	514/559 (91.9%)	69/72 (95.8%)	445/487 (91.4%)	2.17	0.21 (0.65–7.20)
**Preterm (<37 weeks gestational age) delivery**	136/536 (25.4%)	19/71 (26.8%)	117/465 (25.2%)	1.09	0.77 (0.62–1.91)
**NICU stay >4 days**	293/514 (57.0%)	39/66 (59.1%)	254/448 (56.7%)	1.10	0.71 (0.65–1.87)
**NICU complications**	195/516 (37.8%)	27/65 (41.5%)	168/451 (37.3%)	1.20	0.51 (0.71–2.03)
***CHD (with or without surgery) versus no CHD***	320/531 (60.3.%)	33/69 (47.8%)	287/462 (62.1%)	0.56 ^a^	0.02 (0.34–0.93)
**CHD with surgery versus CHD without surgery**	99/320 (30.9%)	9/33 (27.3.%)	90/287 (31.4%)	0.82	0.63 (0.37–1.84)
**CHD with surgery versus no CHD**	99/310 (31.9%)	9/45 (20.0%)	90/ 287 (31.4%)	0.49	0.07 (0.22–1.05)
**CHD without surgery versus no CHD**	221/432 (51.2%)	24/60 (40.0%)	197/372 (53.0%)	0.59	0.06 (0.34–1.03)
**Atrioventricular canal defect**	96/554 (17.3%)	12/71 (16.9%)	84/483 (17.4%)	0.97	0.92 (0.50–1.88)
**Patent foramen ovale**	74/554 (13.4%)	8/71 (11.3%)	66/483 (13.7%)	0.80	0.58 (0.37–1.75)
**Patent ductus arteriosus**	81/552 (14.7%)	7/71 (9.9%)	74/481 (15.4%)	0.60	0.22 (0.27–1.36)
**Atrial septal defect**	113/554 (24.0%)	8/71 (11.3%)	125/483 (25.9%)	0.36 ^a^	0.009 (0.17–0.78)
**Ventricular septal defect**	114/553 (20.6%)	13/71 (18.3%)	101/482 (21.0%)	0.85	0.61 (0.45–1.60)
**Tetralogy of Fallot**	21/554 (3.8%)	5/71 (7.0%)	16/483 (3.3%)	2.21	0.13 (0.78–6.24)
**Respiratory problems**	250/544 (46.0%)	33/72 (45.8%)	217/472 (46.0%)	0.99	0.98 (0.60–1.64)
**Congenital hypothyroidism**	92/536 (17.2%)	10/70 (14.3%)	82/466 (17.6%)	0.78	0.49 (0.38–1.23)
**Acquired hypothyroidism**	82/535 (15.3%)	8/70 (11.4%)	74/465 (15.9%)	0.68	0.33 (0.31–1.48)
**Hyperthyroidism**	17/535 (3.2%)	3/69 (4.3%)	14/466 (3.0%)	1.47	0.55 (0.41–5.24)
***Gl problems***	337/544 (61.9%)	55/72 (76.4%)	282/472 (59.7%)	2.18 ^a^	0.008 (1.23–3.87)
**Constipation**	249/544 (45.8%)	45/72 (62.5%)	204/472 (43.2%)	2.19 ^a^	0.003 (1.31–3.65)
**Gastroesophageal reflux**	145/541 (26.8%)	28/72 (38.9%)	117/469 (38.9%)	1.91 ^a^	0.01 (1.14–3.21)
**Celiac**	31/544 (5.7%)	5/72 (6.9%)	26/472 (5.5%)	1.28	0.63 (0.48–3.45)
**GI surgery**	51/414 (12.3%)	10/50 (20.0%)	41/364 (11.3%)	1.97	0.08 (0.92–4.23)
**Duodenal atresia/stenosis**	27/543 (5.0%)	4/72 (5.6%)	23/471 (4.9%)	1.15	0.81 (0.38–3.41)
**Hirschsprung’s disease**	12/542 (2.2%)	1/72 (1.4%)	11/470 (2.3%)	0.38	0.61 (0.07–4.62)
**Gastrostomy tube**	64/541 (11.8%)	10/68 (14.7%)	54/473 (11.4%)	1.34	0.43 (0.65–2.77)
**Dysphagia without aspiration**	29/541 (5.4%)	4/69 (5.8.%)	25/472 (5.3%)	1.10	0.86 (0.37–3.26)
**Dysphagia with aspiration**	54/542 (10.0%)	8/69 (11.6%)	46/473 (9.7%)	1.22	0.63 (0.55–2.70)
**Special dietary needs**	84/542 (15.5%)	14/69 (20.3%)	70/473 (14.8%)	1.47	0.16 (0.77–2.78)
**Behavioral feeding problem**	22/541 (4.1%)	6/69 (8.7%)	16/472 (3.4%)	2.71 ^a^	0.04 (1.02–7.19)
**Cervical spine problem/atlantoaxial instability**	22/540 (4.1%)	4/68 (5.9%)	18/472 (3.8%)	1.58	0.42 (0.52–4.81)
**Scoliosis**	30/540 (5.6%)	8/68 (11.8%)	22/472 (4.7%)	2.73 ^a^	0.02 (1.16–6.40)
**Hearing loss**	216/547 (39.5%)	31/70 (44.3%)	185/477 (38.8%)	1.25	0.38 (0.76–2.08)
**Hearing aids**	87/547 (15.9%)	9/70 (12.9%)	78/477 (16.4%)	0.75	0.45 (0.36–1.58)
**Eustachian tube dysfunction**	153/547 (28.0%)	18/70 (25.7%)	135/477 (28.3%)	0.88	0.65 (0.50–1.55)
**Tympanostomy ubes**	248/547 (45.3%)	29/70 (41.4%)	219/477 (45.9%)	0.83	0.48 (0.50–1.39)
**History of tonsillectomy**	210/544 (38.6%)	28/68 (41.2%)	182/476 (38.2%)	1.13	0.64 (0.67–1.90)
**History of adenoidectomy**	224/546 (41.0%)	30/70 (42.9%)	194/476 (40.8%)	1.09	0.74 (0.66–1.81)
**Other ear, nose, throat surgery**	77/370 (20.8%)	11/47 (23.4%)	66/323 (20.4%)	1.19	0.64 (0.57–2.46)
**Vision problems**	414/544 (76.1%)	49/68 (72.1%)	365/476 (76.7%)	0.78	0.40 (0.44–1.39)
**Transient myeloproliferative disorder**	8/529 (1.5%)	1/66 (1.5%)	7/463 (1.5%)	1.0	1.00 (0.12–8.28)
**Anemia**	35/532 (6.6%)	6/66 (9.1%)	29/466 (6.2%)	1.51	0.38 (0.60–3.78)
**Leukemia**	12/532 (2.3%)	1/66 (1.5%)	11/466 (2.4%)	0.64	0.67 (0.08–5.01)
***Epilepsy***	22/531 (4.1%)	6/68 (8.8%)	16/463 (3.5%)	2.70 ^a^	0.046 (1.02–7.17)
**Infantile spasms**	11/530 (2.1%)	5/68 (7.4%)	6/462 (1.3%)	6.03 ^a^	0.004 (1.79–20.34)
**Generalized seizures**	13/530 (2.5%)	4/68 (5.9%)	9/462 (1.9%)	3.15	0.06 (0.94–10.51)
**Partial seizures**	5/222 (2.3%)	0/25 (0.00%)	5/197 (2.5%)	0.69	0.80 (0.04–12.78)
**Obstructive sleep apnea**	186/539 (34.5%)	22/68 (32.4%)	164/471 (34.8%)	0.90	0.69 (0.52–1.54)
**ADHD**	54/561 (9.6%)	9/72 (12.5%)	45/489 (9.2%)	1.41	0.38 (0.67–3.02)
**Depression**	9/562 (4.2%)	3/72 (4.2%)	6/490 (1.2%)	3.51	0.08 (0.86–14.35)
**Anxiety**	38/561 (6.8%)	5/72 (6.9%)	33/489 (6.7%)	1.03	0.95 (0.43–2.73)
**Regression/catatonia**	17/562 (3.0%)	2/70 (2.8%)	15/490 (3.1%)	0.90	0.90 (0.20–4.04)

*Italics font*: grouped variable

^a^Statistically significant odds ratio

Individuals with DS+ASD had similar odds of having a history of CHD requiring surgery to those with DS only. There was no observed difference in prematurity or NICU complications between groups. Furthermore, there was no difference in rates of autoimmune thyroiditis or celiac disease. There was also no difference in rates of diagnosed co-occurring ND or MH conditions in this cohort, including anxiety and ADHD.

## Discussion

In children with a dual diagnosis of DS+ASD, multiple medical comorbidities including infantile spasms, constipation, scoliosis, and behavioral feeding difficulties were more likely to be present than in children with DS alone, identifying for the first time that higher rates of multiple medical conditions exist in persons with DS+ASD in childhood. This study builds on previous evidence that a history of epilepsy and/or infantile spasms places a child with DS at significantly higher odds of being diagnosed with ASD [17, 26, 27]. Clinicians can use these findings to increase an index of suspicion for certain gastrointestinal, feeding, and neurologic disorders (e.g., seizures) in children with DS+ASD, including when they may present with non-specific symptoms such as worsening behavior.

Individuals with DS+ASD in this study had twice the odds of being male, consistent with previously published, smaller studies [4, 18], and with the well-documented increased incidence of ASD in males in the general population. There are many theories for this sex difference; some point to inherent bias in our diagnostic criteria [28], while others implicate biological factors such as the influence of Y chromosome haplotypes [29], elevated fetal testosterone [30], and prenatal maternal immune activation affecting neural development leading to ASD phenotypes [31]. The widespread use of non-invasive prenatal testing offers an opportunity to prospectively study these factors in the DS population.

There is a paucity of data on co-occurring medical conditions in DS+ASD. One prior study (with a much smaller sample size) noted an increased prevalence of seizure disorders but failed to identify additional differences in rates of co-occurring conditions between children with DS and DS+ASD [17]. In the present study, children with DS+ASD had significantly higher odds of a diagnosis of gastroesophageal reflux and constipation, consistent with findings in idiopathic ASD [32, 33]. For children with idiopathic ASD, multiple hypotheses have been proposed for the increased frequency of GI complaints, including disruption of the gut-brain axis and alteration in the microbiota [34]. The shared pathophysiological links between GI disturbances and ASD are an area of active research. Further investigation is needed to determine if gut biomarkers studied in individuals with idiopathic ASD [35] are also found with higher prevalence in individuals with DS+ASD. Scoliosis was found to be more prevalent in the DS+ASD group. This was an unanticipated finding that has not been previously reported in the literature and does not have a clear explanation. The association could be indicative of a shared neurological origin for autistic features and neuromuscular weakness.

While several findings in this study are consistent with what has been described in the idiopathic ASD population, there are a few key differences to highlight. Idiopathic ASD occurs with increased frequency in patients with a history of CHD [15], including those with atrial septal defects, while individuals with DS+ASD in this study had lower odds of having a history of either. In this study, rates of prematurity were not significantly different between the two groups, while it is well-documented that prematurity increases the risk of idiopathic ASD [36]. Assessment of the true impact of CHD on the development of ASD is likely underpowered in this single-center design and may be best elucidated in a multi-center study. Additionally, while a variety of MH/ND conditions occur with an increased prevalence in individuals with idiopathic ASD as compared with the general population [37] and prior studies have found increased incidences of impulsivity, ADHD, and anxiety symptoms in patients with DS+ASD [6, 18, 38], this study did not find a statistically significant difference in the frequency of anxiety, ADHD, or depression between the two groups. This may be due to the small number of individuals with these diagnoses among our study participants, and further research is needed.

There was a significant difference in ages between the two groups, with the DS group being younger than the DS+ASD group. However, the difference was small (1.5 years) and could have been a function of excessive power in the DS-only group (*N*= 490 versus *N*=72 in the DS+ASD group). We feel that the age difference found between the two groups is not clinically significant, as its impact on rates of acquired conditions is likely minimal, and it does not affect the rates of congenital conditions.

This study is not without limitations, and the following should be considered when interpreting our findings. Generalizability is affected by the fact that the data was collected from a single tertiary care center. This study relied on parental reports of medical history, which may have been influenced by recall bias at the time of assessment. As such, some respondents left fields blank, limiting the ability to definitively assess if a patient had or did not have a particular condition. This was potentially remedied by obtaining data from multiple encounters and utilizing an “if ever” approach to the conditions (especially as some may resolve). This study design is not conducive to determining whether there is a causal relationship between any of the conditions found to be more prevalent in the DS+ASD group and an ASD diagnosis, and prospective studies in this field are needed. Although the BCH DSP has one of the highest volume pediatric DS centers in the country, ASD is only present in a minority of persons with DS and thus this study may have been underpowered to detect more nuanced medical contributions to ASD in children with DS, specifically in more rare conditions. This is further compounded by the fact that some medical conditions, such as autoimmune conditions, develop later in childhood and thus may have not yet been diagnosed in some of the younger patients included in this study. Future research should employ data gathered from a clinician using a systematic method to obtain a more complete picture of medical conditions impacting individuals with DS. Ongoing efforts supported by the National Institute of Health, like the Human Trisome Project, a pan-omics cohort study with deep matching clinical data, and DS-Connect®, a secure, web-based registry, are promising databases that may help further elucidate how children with DS+ASD differ from those with DS alone.

## Conclusion

This study identifies a variety of medical conditions that are more frequent in children with DS+ASD than DS alone. Results from this work support prior literature on DS+ASD and epilepsy and further our understanding of co-morbidities in the DS+ASD population. Findings from this study can inform the clinical management of individuals with DS+ASD by prompting early and routine screening for conditions identified as more prevalent. Future research will need to expand on these findings and investigate whether there are distinct biologic contributions towards these conditions in children with DS+ASD.

## Supplementary Information

**Additional file 1.** Explanation of Grouped Variables.

## Data Availability

The datasets used and/or analyzed during the current study are available from the corresponding author on reasonable request.

## Abbreviations

ASD: Autism spectrum disorder
DS: Down syndrome
DS+ASD: Down syndrome-autism spectrum disorder
ND: Neurodevelopmental
MH: Mental health
DSM: Diagnostic Statistical Manual
ADOS: Autism Diagnostic Observation Schedule
CHD: Congenital heart disease
GI: Gastrointestinal
DSP: Down Syndrome Program
REDCap: Research Electronic Data Capture
EMR: Electronic Medical Record
NICU: Neonatal Intensive Care Unit
ADHD: Attention deficit hyperactivity disorder
OR: Odds ratio
IQR: Interquartile range
