# Supplementary material for: Co-occurring conditions in children with Down syndrome and autism: a retrospective study
Source: J Neurodev Disord. 2023 Mar 2;15:9. doi: 10.1186/s11689-023-09478-w (PMC9979529; doi:10.1186/s11689-023-09478-w)
Supplement: Supplementary file 1 — Additional file 1. Explanation of Grouped Variables. [file 11689_2023_9478_MOESM1_ESM.docx]

**Additional file 1: Explanation of Grouped Variables**

| **Congenital Heart Disease** | Congenital heart disease with history of surgery |
| --- | --- |
|  | Congenital heart disease without history of surgery |
| **GI Problems** | Constipation |
|  | Gastroesophageal Reflux |
|  | Celiac disease |
|  | GI surgery |
|  | Duodenal Atresia/Stenosis |
|  | Hirschsprung's Disease |
| **Epilepsy** | Infantile Spasms |
|  | Generalized Seizures |
|  | Partial Seizures |
